# Southern Branch, National Dental Association, Second Annual Meeting, New Orleans, La., February 9, 10, 11, and 13, 1899

**Published:** 1899-05

**Authors:** 


### PROCEEDINGS.

### Southern Branch, National Dental Association, Second Annual Meeting, New Orleans, La., February 9, 10, 11 and 13, 1899.

[*Continued from April No., Page* 180.]

### "OPERATIVE DENTISTRY."

BY J. G. FIFE, DALLAS, TEXAS.

In his journal review of the subject Dr. Fife said that all energies were being devoted to the substitution for gold of that which would be less conspicuous; to improve the quality of and the manner of manipulating and inserting amalgams; to produce a stable cement; to improve upon unscientific methods in the treatment and filling of root canals; to ascertain the etiology of pyorrhea alveolaris and to discover a line of treatment that will eradicate this fell destroyer of the dental organs; in fact, to elevate dentistry to the highest scientific plain, and the dentist to the highest type of manhood. The most prominent movement is toward the development of the artistic. The literature of the years bears evidence of profound and minute investigation. The report concluded with a list of some the best papers which have appeared during the past year.

DISCUSSION.

DR. MCKELLOPS expressed his renewed surprise that so little attention is given to the use of platinum-gold for the anterior teeth—so little that it was with difficulty the manufacturers could be induced to continue its manufacture, and yet it is the ideal filling material for the front teeth. He said, *Why* don't you use it, and do yourself, your profession and your patients the most good?

Dr. H. A. Smith spoke of the new oblundent vapocain, and the theory of this new etherial solution of cocaine.

Dr. T. P. Hinman has had good results from vapocain in five minutes in a very sensitive cavity. Dr. Hinman is working the Jenkins' system of porcelain inlays with very fair results.

Dr. Wm. V. B. Ames has not been favorably impressed with porcelain inlays. He thinks that where a porcelain inlay can be used once, gold inlays can be used nine times. He said that with the ability of a Dr. McKellops nothing could equal platinum-gold, nothing could equal that material for the anterior teeth.

Following the advice given by Dr. McKellops in 1875, Dr. T. M. Allen has been using platinum-gold ever since, and having the choice of three shades it is possible to so fill anterior teeth that the fact of its being a gold filling is not perceptible. Corners can be perfectly built out and it resists the wear of mastication perfectly.

Dr. L. A. Smith, Port Gibson, Miss., read a paper on

"THE ADVANTAGES OFFERED IN A COMBINATION OF DIFFERENT FILLING MATERIALS,"

and the effect of different filling materials upon the tooth itself, the deposits of secondary dentin bearing some analogy to the encysting of foreign bodies by the soft tissues. He has not had favorable results from the combination of tin and gold.

Dr. J. Y. Crawford spoke very strongly against the use of amalgam, either singly or in combination. In the use of tin and gold he has recently been reversing the usual method and uses gold next to the tooth substance, especially at the cervical walls, where it does not turn dark as does the tin usually placed there. He believes that the secondary deposits are made in spite of, rather than because of the presence of an irritating filling. The treatment of the teeth may bring about conditions which stimulate powers of resistance in the teeth themselves by which their subsequent integrity is maintained.

### NOTES ON MATERIA MEDICA, WITH COMMENTS AND CRITICISMS.

BY H. H. JOHNSON, D.D.S., MACON, GA.

He spoke of the many new and wonderful chemical combinations that are being brought to our attention, the dental supply houses not being entirely exempt from criticism in this respect, new appliances and preparations being often pushed forward before they have been fairly tested or their practical value proved. He spoke of the widely diverging theories in regard to the use of coagulants, one party claiming that the coagulated albumen prevents the diffusion of the medicament—the other side claiming that the coagulum is redissolved after being formed and the agent readily diffused. The only way out of the difficulty is to assume that either theory may be correct, since both appear to be equally well tested, and use our own judgment. Those who in the past relied upon carbolic acid and creasote certainly saved many teeth in spite of these coagulants. Dr. Johnson concludes that possibly it was because the mechanical preparation was so perfect that there was not very much albuminous matter left to be coagulated, assuming, of course, that the coagulum would be a barrier to diffusion.

Recent writers on formaldehyde were quoted, the conclusion reached being that it is too intensely irritating to be used in sufficient strength to have the value of other well-known and tested antiseptics. Eucaine as a substitute for cocaine was favorably considered. Milk of magnesia was highly commended.

DISCUSSION.

Dr. Barrett spoke very forcibly in favor of continuing the use of the old and tried remedies which have done good in the comparison with the newer chemical combinations with which we are being overloaded by the manufacturers. He said, do not let us abandon these old and tried remedies; the claims made for the newer agents are not made upon clinical experience but are based upon theory. Relying upon the results of personal experience may not be so scientific, but it is eminently satisfactory.

Dr. Crawford spoke of the value of carbolic acid in the treatment of abrasion or erosion. Carbolic acid not only reduces the sensitiveness but holds erosion in check. It is also valuable in the treatment of root canals, aiding nature in closing up the apical foramen.

Dr. H. A. Smith spoke of the theory of Dr. A. C. Hart, of California, as to the value of formaldehyde as a prophylactic in dental caries, and as silver nitrate has been used but with the advantage of being free from discoloration. In a case where the dentin seemed to be dissolving out from under the enamel, the application of formaldehyde apparently arrested the action. While it is not well to try every new thing that comes up, many of them are good and what we need.

Dr. L. M. Cowardin said he had only an empirical knowledge of formaldehyde, but it appeared to him to be rather a remarkable agent. Experimenting with a view to a formula for an antiseptic mouth-wash he had found that one-third to one-half of 1 percent of the 40 percent solution, as we get it, added to the value of the mouth-wash in relieving sensitiveness at the cervical borders. It rapidly deodorizes putrescent root canals and the odor does not return, but it is also apt to produce apical irritation.

Dr. S. W. Foster spoke of the value of iodin in the treatment of root canals when used as a vapor, by means of Blair's Vaporizer; it is especially valuable after treatment with the essential oils.

Dr. B. Holly Smith does not think that formaldehyde in the 40 percent solution is applicable to the treatment of live teeth, especially when deprived of enamel. He thinks too much prominence has been given to the theory of Dr. Hart. He thinks almost any other agent would have about as much effect in a mouth-wash as one-third of 1 percent of the 40 percent solution of formalin.

---

Dr. C. V. Vignes, New Orleans, La., read a paper on

### THE TREATMENT OF PULPLESS TEETH BY IODOFORM FUMES UNDER PRESSURE, using the Blair Vaporizr. Dr. Vignes detailed the method of treating pulpless teeth under the three classes—a dead pulp that has given no trouble; with fistulous opening through the gum, and so-called blind abscess. Dr. Vignes, after an experience of nine years with the vaporizer, claims that by this method there can be accomplished with certainty that which requires patience and perhaps several weeks of tedious work to accomplish by any other method.

DISCUSSION.

Dr. T. C. West, finding the odor of iodoform so uniformly objectionable, had experimented in other directions, and has obtained very good results from the use of acetanelid in the vaporizer, and has been using it for several years. As a deodorizer for root canals it is perfect.

Dr. Barrett said that he would like to know what special quality iodoform has that it should be considered infallible; that it should *always* give good results. He could not conceive that any single remedy could produce such uniform results, penetrating infallibly all the tissues. Iodin is undoubtedly beneficial in many cases, but he cannot conceive of such wonderful results as are attributed to it by the essayist.

Dr. Vignes, in closing the discussion, said that he could only speak for the uniform results attained in an experience of nine years. He had not tried acetanelid. He had tried sulphur, but could not control the heat with sufficient accuracy.

Operative dentistry passed.

### CHEMISTRY, METALLURGY AND ANATOMY.

BY DR. A. B. KING, BALTIMORE, MARYLAND, CHAIRMAN.

He reported great progress made during the past year in the discovery of new elements, no less than six having been added to the list, though the details in regard to some of them are so meagre that there is perhaps reason for doubt as to their actual existence as elements. Three of the new elements, Krypton, Neon and Metargon, were discovered by Prof. Wm. Ramsey, and found with argon, after it had been isolated from liquid air. Polonium is in the form of a sulphide of pitchblende, resembling bismuth chemically, and with a radiating power 400 times that of branium, the latter element throwing off an invisible radiance much like that of the Roentgen rays. Caronium has thus far been shown only by the spectroscope, and only in the solar atmosphere, though at a distance of 300,000 miles from the sun's surface. Etherion is an element which Mr. Charles F. Brush, the well-known electrician, thinks he has discovered in the earth's atmosphere. Its heat-conductivity is 100 times as great as that of hydrogen.

The work upon the proteids, their decomposition and digestion products, has been exhaustively studied during the past year. Of especial interest are the studies by Kutscher upon the body named by Kuhne, antipeptone.

In anatomy the most important work of the year is the paper by Dr. G. V. Black, in the *Dental Cosmos* for February, 1899, in which the author claims to have discovered a series of glands in the peridental membrane, with ducts leading to the gingival margin.

### DEFECTIVE TOOTH DEVELOPMENT.

BY DR. L. M. COWARDIN.

The patient, who is nearly fifteen years of age, has the temporary superior laterals and canines still in place, with no sign of absorption of the roots or of the approach of the permanent teeth. The first temporary right superior molar has been replaced by a well-formed canine; the second right superior temporary molar is still in place with nothing to indicate the approach of a bicuspid. The left superior temporary molars were lost two or three years ago, but there is no intimation of the approach of the bicuspids. Both right and left first and second superior permanent molars are in place and well formed. The inferior permanent incisors and canines erupted at the usual time. With the exception of one, the temporary inferior molars are in place, with no sign of approaching bicuspids.

The papers were passed without discussion.

DR H. H. JOHNSON, Macon, Ga., Chairman of the Committee, presented as his report a subject-index of the journalistic literature of the past year upon

PATHOLOGY, MATERIA MEDICA AND THERAPEUTICS, which was ordered published in the volume of Transactions, as being of great value for reference.

### "NOTES ON MATERIA MEDICA, WITH COMMENTS AND CRITICISMS."

BY H. H. JOHNSON, D.D.S., MACON, GA.

The paper was designed to call attention to a few new preparations, and to revive interest in some older ones which have been to some extent displaced by new ones possessing less meritorious points. Dr. Johnson quoted as follows from a London publication: "Medicine is swamped, drowned, stifled and paralyzed by innumerable exploiters, within and without its ranks; exploiters whose only object is the shortest possible cut, not to fame and fortune, but to notoriety and pelf." This is as applicable to dentistry as to medicine, with the result that the average mind is either suffocated with what appears to be new knowledge, or the practitioner makes up his mind on no earthly consideration to experiment with a new drug.

In dental materia medica, antiseptics occupy, probably, the most important position of all the medicines used, and demand the most careful study and discrimination. Theory and practice are widely divergent in actual results obtained; an artificially demonstrated theory cannot be accepted until it has been proven by passing through the experimental stage.

Dr. Johnson then reviewed briefly the "coagulation" and "anti-coagulation" theories in regard to the treatment of septic conditions existing in devitalized teeth, reaching the conclusion that as both theories are apparently equally well sustained, it is safe to assume that either may be correct and go on saving teeth as of old by the use of carbolic acid, creasote, etc.

Dr. Johnson described the value, properties and uses of other well-known remedies—aromatic sulphuric acid, trichloric acetic acid, iodin and others.

Of the newer remedies, Dr. Johnson, while quoting from other writers as to the value of *Formalin*, is not himself favorably impressed with this drug, because of its intensely irritating qualities. With *Eucaine* as a substitute for cocaine he is most favorably impressed.

DISCUSSION.

DR. BARRETT spoke very forcibly in favor of the old and tried remedies that have stood the test of time. He said the use of carbolic acid in the treatment of devitalized teeth has been decried because it is a coagulant, but it derives its chief value from being a coagulating agent. The first step necessary in the removal of the albuminous contents of the dentinal tubules is coagulation, which is the first step in the melting-down process. Tincture of iodin is another old stand-by, especially for injection into root canals when there is tumefied induration, because it promotes absorption. Tincture of aconite is another reliable remedy, constant in its action upon terminal blood-vessels. Dr. Barrett said we all need to understand the remedies we use, and should know how to prescribe them intelligently, according to the pathological conditions existing. The time is coming when the diseases of the pharynx and other territories contiguous to the oral cavity will be acknowledged to lie within the purlieus of dentistry. Who has a better opportunity of seeing down the throat than the dentist? He should, therefore, be competent to read aright the signs, recognizing all diseased conditions and prescribing therefor.

DR. J. T. CRAWFORD spoke of the value of carbolic acid in the treatment of erosion, not only relieving the sensitiveness of the polished abraded surfaces but retarding the progress of erosion, holding it in check. Dr. Crawford gives this as the result of observations extending over a number of years: After the removal of a pulp, the use of carbolic acid in the canals will aid nature in closing up the apical foramen. There is nothing like sterilized coagulated albumen for the protection of the periosteal or pericemental tissues.

Dr. H. A. Smith, Cincinnati, spoke of the theory recently advanced by Dr. Hart, of California, as to the value of formalin as a prophylactic in dental caries, on the same principle that silver nitrate has been used, formalin being free from the objection to silver nitrate, that of staining. Dr. Smith also spoke of the new obtundent vapocain, and the theory of its action.

Dr. T. P. Hinman, Atlanta, Ga., has been using the vapocaine with fairly good results; in five minutes, in one case, in which the dentin was very sensitive.

Dr. L. M. Cowardin uses one-half or one-third of 1 percent of the 40 percent solution of formalin as a constituent in a mouth-wash, with very beneficial effects, personally, for sensitiveness at the cervical portions of the teeth. If the use of the wash is discontinued for a few weeks the sensitiveness returns. This is the result of two years' experience with the mouthwash. Its action is due to its powerful coagulating effect, hardening the organic matter of the tooth. Dr. Cowardin uses it in root canals in 10 and 15 percent strength. It rapidly deodorizes putrescent root canals, but is apt to produce apical irritation unless used with great care.

Dr. S. W. Foster, Atlanta, Ga., spoke of the value of cocain in pulp devitalization, and hopes that arsenious acid will be entirely discarded. He spoke of the value of iodoform vapor, pumped into the tissues, and the use of sulphuric acid in enlarging the canals.

Dr. B. Holly Smith thinks formalin not suited to use in the living tooth, especially one deprived of enamel. It is liable to cause serious pulpitis, difficult to subdue.

Dr. C. V. Vignes, New Orleans, read a paper on the treatment of pulpless teeth with the fumes of iodoform, using Blair's vaporizer. Iodoform is sublimated at a temperature of about 240 degrees, F. In cooling the vapor condenses into iodoform minus a small percentage of iodin that is set free. Being very penetrating when in its vapor state, under pressure it goes to every part of the tooth under treatment, even into the tubuli, rendering asceptic every portion with which it comes in contact, crystalizing against the walls of the root canal to the opical foramen. For nine years Dr. Vignes has had unvarying success with this method of treating pulpless teeth, whether with fistulous opening or "blind abscess." When it is not possible to remove all the contents of the root canals by this treatment, the remnants are so dessicated and encased in iodoform as to be rendered inoffensive.

Dr. T. C. West, Natchez, Miss., has for a number of years used acetanelid with the Blair vaporizer, with very happy results, proving to be a perfect deodorizer while free from the objectionable odor of iodoform.

Dr. W. C. Barrett is at a loss to see what special quality iodoform possesses that it can be regarded as infallible, *always* giving good results. While iodin is indubitably beneficial in many cases, he fails to see what there is in its action to produce such wonderful results.

Subject passed.

An interesting discussion followed the presentation of the following resolution by Dr. B. Holly Smith, of Baltimore, and unanimously adopted by the Association:

"Whereas, the Southern Dental Association recognizes the fact that under the loosely-drawn statutes of some of the States of America it has been and still is possible to incorporate fraudulent dental and other colleges, and to obtain the certificate of the officers of state testifying to the legal regularity and status of institutions known to be openly selling different diplomas abroad without pretense of instruction; and

"Whereas, we further recognize that such vending of our degree by such fraudulent colleges, and the wholesale flooding of European countries with counterfeit diplomas has brought discredit upon the distinctive American degree of dental surgery and has induced special legislation against it in such countries and made of it a badge of dishonor rather than honor in the estimation of many educated people; and

"Whereas, the good name of the dental profession of America is at stake, and we are held to our full share of responsibility for this condition of our confreres abroad; therefore be it

"*Resolved*, by the Southern Dental Association, in annual meeting assembled, That we will as a society and individual members of the profession, do all that is within our power to obtain the repeal of any acts under which it is possible for irresponsible dental and other schools to become incorporated, and that we will spare neither time, labor nor money in the suppression of those now in existence, and in bringing to condign punishment the villains engaged in connterfeiting our dental diplomas.

"*Resolved*, That we heartily commend the action of the Foreign Relations Committee of the National Association of Dental Faculties in unearthing and making public the existing condition, and that we will sustain them to the utmost extent of our ability in their efforts to put a stop to the traffic in fraudulent diplomas."

During the discussion of this resolution, Dr. Barrett, of Buffalo, made a most forceful and telling argument against certain institutions that make a practice of selling diplomas and degrees, especially in the city of Chicago. They have flooded the whole of Europe with their circulars, until the American professions have come to be regarded with contempt among the European scientists and professional men. He urged that some drastic measures should be taken for the suppression of institutions that are a disgrace to the whole country.

Dr. McKellopps, of St. Louis, said that there are institutions, of the kind referued to by Dr. Barrett, in Kansas City also.

After a full discussion of the subject and the adoption of the resolution offered by Dr. Smith, the association took a recess until 5 o'clock.

At the next session the first order of business was the report of the Committee on the Appointment of Dental Surgeons in the Army and Navy.

Dr. B. Holly Smith, of Baltimore, as a member of the committee, stated that there was nothing to report, as the body had never been called together by the chairman. Dr. Smith then offered a number of suggestions. He said that the matter should be taken up and now was the time for action—to strike while the iron was hot. To put the matter off would be fatal to the prospects of accomplishing anything. The members present should communicate with their representatives in Congress and urge upon them the necessity of having dental surgeons in both the army and navy.

Dr. McKellopps, of St. Louis, arose after Dr. Smith had concluded his remarks and said that even if such a bill was passed by Congress the examination the students would be subjected to would be so rigid that few if any could pass it. He was told this by one of the physicians of the army. The student, he contended, would have to be thoroughly efficient in both dentistry and medicine.

Dr. B. Holly Smith said that if a dentist had received a college education or otherwise, and graduated therefrom, the chances were that he would have no trouble in passing the government examination.

Dr. Morgan, of Nashville, said he entertained great hopes for the successful passage of the bill providing for dentists in the army if sufficient influence was brought upon the Committee of Conference of the House. The matter should be brought immediately before that body.

Dr. Hinman, of Atlanta, said that if the members made the proper efforts Congress would see the necessity of passing a bill to that effect. The Committee on Dentists in the Army and Navy having done nothing on account of the time not being propitious, Dr. Smith asked that the committee be discharged.

Dr. Crawford moved that the committee be continued and the secretary instructed to write the chairman and ask that the body be called together for the purpose of having them bring the matter before the House of Representatives again. Dr. Crawford was heartily in favor of instituting a vigorous campaign. It became an imperative duty for Congress to pass a bill that meant so much to soldiers and sailors. The speaker told of the suffering of UncleSam's men at Camp Wyckoff and on board the transports coming from Cuba. The suffering caused by the teeth was something terrible.

Dr. T. M. Allen, of Alabama, spoke on the subject, as did also Dr. Laurence Leonard, of Waseca, Minn.

As an amendment to Dr. Crawford's resolution, Dr. V. E. Turner, of North Carolina, suggested that the different State societies be notified of the convention's action and they be requested to co-operate with the committee and use their influence for the speedy passage of a bill for the appointment of dentists in the army and navy.

The resolution, with the amendment, was carried.

At a later session, as voluntary essays, Drs. Wallace Wood, Jr., and Laurence Leonard presented papers entitled "Dentists in the Army and Navy." The papers showed that it was absolutely necessary for Congress to make some provision for the appointment of dental surgeons in the army and navy. The matter was of vital importance, they contended, and with proper urging on the part of the various State societies and the Southern and National Dental associations and the individual members themselves, Congress would recognize the importance and pass a bill to that effect. The matter of dental surgeons in the army and navy having been generally discussed at the second day's meeting of the association, the papers were passed without further discussion.

Dr. H. B. Smith, in a very touching manner, said that it was his painful duty to inform the convention of the misfortune which had befallen Dr. Truman W. Brophy, of Chicago, one of their colleagues in the death of his wife. Dr. Brophy was to have read a paper and to have given a clinic, operating upon cleft palate, but the sad occurrence prevented him from coming South.

Dr. Holly B. Smith offered a resolution tendering to Dr. Brophy in the hour of his bereavement the deepest sympathy of the convention. The resolution was adopted by a rising vote and was telegraphed to Dr. Brophy at Chicago.

Dr. Noel read a paper which had been sent to the convention by Dr. Beadles, of Danville, Va., ex-president of the Southern branch of the National Dental Association. Dr. Beadles stated that he had prepared to come to this convention and was very anxious to be in attendance, but just before he was to start for New Orleans he was summoned on the jury and the judge of the Danville court compelled him to serve, refusing to recognize dentists as coming in under the immunity and exemption from jury duty. He urged that this matter be taken up for consideration by the association and that some action be taken to positively determine and define whether dentists are professional men or mechanics. The matter was discussed at great length, statements from members present showing that in many of the States represented in the convention the dentists are exempt from jury duty. Dr. Cowardin, of Richmond, desired to have it distinctly understood that the action against Dr. Beadles, in Danville, is that merely of an individual judge, and must not be taken as a reflection upon the whole State of Virginia, for in all other portions of the State dentists are not required to serve on juries.

